# Widespread anorectal lymphovascular networks and tissue drainage: analyses from submucosal India ink injection and indocyanine green fluorescence imaging

**DOI:** 10.1111/codi.15582

**Published:** 2021-03-01

**Authors:** Kentaro Sato, Hiroshi Shimoda, Takuya Miura, Yoshiyuki Sakamoto, Hajime Morohashi, Seiji Watanabe, Hirokazu Narita, Yuto Mitsuhashi, Kotaro Umemura, Kenichi Hakamada

**Affiliations:** ^1^ Department of Gastroenterological Surgery Graduate School of Medicine Hirosaki University Hirosaki Aomori Japan; ^2^ Department of Anatomical Science Graduate School of Medicine Hirosaki University Hirosaki Aomori Japan; ^3^ Department of Neuroanatomy, Cell Biology, Histology Graduate School of Medicine Hirosaki University Hirosaki Aomori Japan

**Keywords:** anal canal, anatomy, lymphovascular network, levator ani muscle, indocyanine green

## Abstract

**Aim:**

Abdominoperineal resection is associated with poor prognosis in patients with advanced lower rectal cancer. This study aimed to analyse the functional lymphovascular network and tissue drainage in the anorectal region.

**Methods:**

In this descriptive study, we performed microanatomical evaluations and intra‐operative imaging analysis in a cadaver and patients with rectal cancer. Specimens with India ink injection were collected from a cadaver and from six patients who underwent abdominoperineal resection. Intra‐operative indocyanine green fluorescence imaging was performed on four patients who underwent surgery for lower rectal cancer. India ink was injected into the submucosa at the dentate line of specimens. Tissue sections were examined by immunohistochemistry for D2‐40 and CD31. Intra‐operative indocyanine green was injected into the submucosa at the dentate line. Lymph flow was traced using a near‐infrared camera system.

**Results:**

Fascia branching from the rectal longitudinal muscle layer extended to the posterior hiatal ligament and lateral endopelvic fascia connective tissue lamina on the surface of the levator ani muscle. The fascia contained veins labelled with ink in their lumina and initial lymphatics. Intra‐operative indocyanine green fluorescence imaging revealed extensive lymph flow from the muscle layer of the anal canal to the hiatal ligament and endopelvic fascia along the longitudinal muscle layer fibres.

**Conclusions:**

The anorectal region contained widespread venous and lymphatic networks in proportion to its specific connective tissue framework around the longitudinal‐muscle‐layer‐extending muscle bundles, which provides extensive networks for tissue fluid and cells.

**What does this paper add to the literature?**

This study provides a description of microanatomical and functional lymphovascular networks in the anorectal region, which has not been previously reported. This paper may help for developing therapeutic strategies for early and advanced lower rectal cancer.

## INTRODUCTION

Abdominoperineal resection (APR) is a standard operative procedure for advanced lower rectal cancer (RC) located within 5 cm of the anal verge. Sphincter‐preserving procedures (SPPs), such as intersphincteric resection, are considered optional procedures. In cases with T3–4 lower RC, APR and SPPs demonstrate equal circumferential resection margin (CRM) positive rates [1]. Despite the fact that the resection area of APR is larger than that of SPPs, APR is associated with poorer outcomes than SPPs [2, 3, 4]. To improve prognosis, extralevator abdominoperineal excision (ELAPE), a more extensive procedure than standard APR, was introduced [5]. ELAPE has been reported to improve oncological outcomes [6, 7, 8, 9] due to its enlarged resection area; however, some studies have reported that ELAPE does not improve outcomes [10, 11, 12]. The reasons for these controversies remain unclear. Thus, examining the lymphovascular network in the anorectal lesion is important as the tumour may spread beyond the APR resection area via these vasculatures. However, a limited number of studies have investigated this from a microanatomical perspective. Most microanatomical studies around the anal canal examine muscular structures [13, 14]. Some studies reported anorectal lymphatic pathways using anatomical lymph node mapping [15, 16]. However, this method can only investigate the location and distribution of lymph nodes and cannot investigate the functional lymphatic flow. Some studies examined the rectal lymphatic flow, including the lateral region, using isotope methods [17, 18, 19], but isotopes were only detected in lymph nodes, not lymphatic ducts, rendering the conclusions of these studies speculative. Besides, the anorectal lymphatic drainage from the levator ani muscle (LAM) and its surrounding fascia has not been investigated yet. Microanatomical examinations of these areas can provide important information to adapt therapeutic strategies for lower RC.

This study aimed to analyse the detailed functional lymphovascular network in the anorectal region. We evaluated the microanatomical organization of lymphovascular communications from data obtained by immunohistochemistry after submucosal India ink injection into fresh cadavers and resected specimens, and from intra‐operative indocyanine green (ICG) fluorescence imaging (FI).

## METHODS

### Analysis of functional lymphatic and vascular networks in the anorectal region

#### Participants

Table 1 presents the patients’ characteristics. The cadaver of a 58‐year‐old woman without intrapelvic lesions, donated to the Hirosaki University Graduate School of Medicine within 12 h after death, and six specimens from six patients (four men and two women; median 68 years, range 57–77 years; tumour distance from the anal verge 4 cm, range 0–6 cm; Stage I–IIIC [20]; three patients received preoperative chemotherapy) who underwent APR at Hirosaki University Hospital between February and August 2019 were included in this study. The study was approved by the Human Research Ethics Committee at Hirosaki University Graduate School of Medicine (Aomori, Japan; reference no. 2017‐1140 and 2018‐134). Informed consent was obtained from all participants.

**TABLE 1:** Characteristics of participants of India ink injection

Case	Fresh cadaver or patient with RC	Age (years)	Sex	BMI (kg/m^2^)	Distance from AV (cm)	Preoperative chemotherapy	Operation	Operative time (min)	Bleeding (ml)	Pathology	Tumour size (cm)	pT	pN	pM	pStage
1	Fresh cadaver	58	F	–	–	–	–	–	–	–	–	–	–	–	–
2	Patient of RC	57	M	20.2	4	SOX+bevacizumab	APR	142	30	Adenocarcinoma‐well	6.5 × 5.0	2	0	0	I
3	Patient of RC	59	M	24.5	0	None	APR	375	30	Adenocarcinoma‐well	4.5 × 3.5	2	0	0	I
4	Patient of RC	70	M	20.8	6	SOX+bevacizumab	APR	330	50	Adenocarcinoma‐mod	5.0 × 5.0	2	0	0	I
5	Patient of RC	77	F	24.9	5.5	None	APR	348	50	Adenocarcinoma‐well	2.0 × 2.0	3	0	0	IIA
6	Patient of RC	77	F	25	0	None	APR	347	0	Adenocarcinoma‐well	5.5 × 3.5	4b	1a	0	IIIC
7	Patient of RC	66	M	23.5	4	SOX+bevacizumab	APR	563	280	Adenocarcinoma‐mod	4.0 × 4.0	3	0	0	IIA

Abbreviations: Adenocarcinoma‐mod, moderately differentiated adenocarcinoma; Adenocarcinoma‐well, well differentiated adenocarccinoma; APR, abdominoperineal resection; AV, anal verge; BMI, body mass index; RC, rectal cancer; SOX, S‐1 and oxaliplatin.

#### India ink injection and microanatomical analyses

India ink (1 ml; Kuretake, Nara, Japan) was injected into the submucosa at the anterior, posterior and bilateral walls on the dentate line (3 cm oral side from the anal verge) of the specimens. In the study using surgical specimen tissues, lesions occupied by tumours were excluded from injection of India ink.

The cadaver was fixed in 10% formalin by arterial perfusion and routinely embalmed on the day after India ink injection. The anorectal tract and its surrounding tissues, including the internal anal sphincter, external anal sphincter (EAS) and LAM, were resected en bloc from the cadaver. The posterior and bilateral regions (up to 7 cm oral side from the anal verge) with the India ink injection were cut along the sagittal and frontal planes, respectively. The surgical specimens, excluding tissues for pathological diagnosis, were immersed for at least 1 day in 10% formalin 1 h after India ink injection and sectioned in the longitudinal plane. All samples were dehydrated in a graded ethanol series, embedded in paraffin, and cut into 5‐μm‐thick sections for haematoxylin–eosin staining, silver impregnation staining and immunohistochemistry. The tissues excluded from pathological diagnosis, which included tumour cells not injected with India ink, were also examined.

#### Immunohistochemistry

After deparaffinization, tissue sections were incubated in a 0.01 m citrate buffer (pH 6.0) at 121°C for 15 min to retrieve the antigenicity of the relevant proteins. The sections were then immersed in 0.3% H_2_O_2_ and 0.1% sodium azide (Wako Pure Chemical Industries) in phosphate‐buffered saline (1/15 m, pH 7.4) for 25 min to block endogenous peroxidase activity. After incubation in 10% normal goat serum (Vector Laboratories), sections were treated with antibodies against podoplanin (1:100, D2‐40; Dako) and CD31 (1:250, EP3095; Abcam) at room temperature overnight. After washing in phosphate‐buffered saline, sections were incubated with alkaline‐phosphatase‐conjugated anti‐mouse IgG (Histofine Simple Stain AP, Mouse; Nichirei Bioscience) for 1 h at room temperature as a secondary antibody for podoplanin immunostaining. The blue immunoreaction was visualized using an alkaline phosphatase reaction (Vector blue substrate kit, Vector Laboratories). Sections were then incubated with peroxidase‐conjugated anti‐rabbit IgG (Histofine Simple Stain MAX‐PO, Rabbit; Nichirei Bioscience) for 1 h at room temperature. The brown immunoreaction for CD31 was visualized using 3,3'‐diaminobenzidine reaction (Wako Pure Chemical Industries). The stained sections were examined using a BX‐60 light microscope equipped with a DP72 digital imaging system (Olympus).

### Intra‐operative indocyanine green fluorescence imaging

#### Participants

Table 2 demonstrates the clinicopathological characteristics of the participants. The specimens were four male patients (median age 58 years, range 47–62 years; tumour distance from the anal verge 5.5 cm, range 3–7 cm; Stage I–IIIB [20]; operative procedures APR (*N* = 1), low anterior resection (*N* = 3); all four patients received preoperative chemotherapy) who underwent laparoscopic or robotic surgery for lower RC at Hirosaki University Hospital between January and March 2019. This study was approved by the Human Research Ethics Committee at Hirosaki University Graduate School of Medicine (reference no. 2017‐044 and 2018‐134). Informed consent was obtained from all participants.

**TABLE 2:** Clinicopathological characteristics of patients undergoing ICG‐IF

Case	Age (years)	Sex	BMI (kg/m^2^)	Distance from AV (cm)	Preoperative chemotherapy	Operation	Operating time (min)	Bleeding (ml)	Pathology	Tumour size (cm)	pT	pN	pM	pStage
1	47	M	25.9	3	FOLFOX + panitumumab	APR	760	880	Adenocarcinoma‐well	8.0 × 8.0	3	2a	0	IIIB
2	60	M	23	7	SOX	LAR	530	130	Adenocarcinoma‐mod	4.6 × 3.7	3	0	0	IIA
3	56	M	18.7	6	SOX	LAR	505	10	Adenocarcinoma‐mod	4.8 × 3.4	3	0	0	IIA
4	62	M	20.3	5	SOX	LAR	513	50	Adenocarcinoma‐mod	3.5 × 3.4	2	0	0	I

Abbreviations: Adenocarcinoma‐mod, moderately differentiated adenocarcinoma; Adenocarcinoma‐well, well differentiated adenocarccinoma; APR, abdominoperineal resection; AV, anal verge; BMI, body mass index; FOLFOX, fluorouracil, leucovorin and oxaliplatin; ICG‐FI, indocyanine green fluorescence imaging; LAR, low anterior resection; SOX, S‐1 and oxaliplatin.

#### Indocyanine green fluorescence imaging and image analyses

ICG solution was prepared in the same manner as described in a previous study evaluating lymphatic flow in the colon [21]. Twenty‐five milligrams ICG powder (Diagnogreen; Daiichi Pharmaceuticals) was dissolved in 10 ml distilled water. The patient was administered general anaesthesia, and then 0.1 ml (0.25 mg) of the dye solution was injected into the submucosa at the dentate line (3 cm oral side from the anal verge) at the anterior, posterior and bilateral walls prior to the initiation of the operation. After completing a total mesorectal excision, the lymph flow around the anal canal was observed using a laparoscopic near‐infrared camera (1588 AIM camera system; Stryker).

#### Illustration of the anorectal functional lymphovascular network

From the collected data, schemas of the functional lymphovascular network of the anorectal region were generated using illustration software, Adobe Illustrator CC (Adobe Systems). Schemas were illustrated according to observations in our study and a previous report by Tsukada et al. [13].

## RESULTS

**FIGURE 1:**
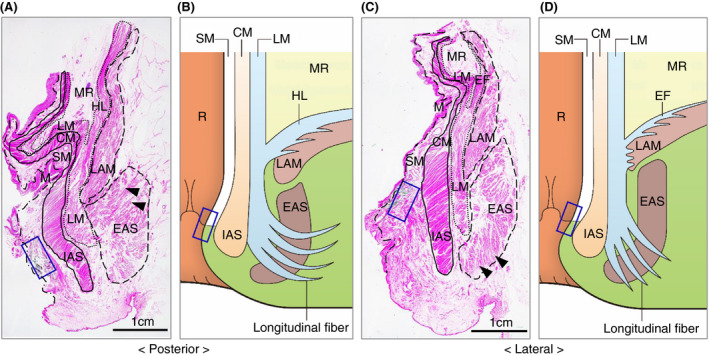
Low‐magnification views of India ink injected specimens. Injection points are shown as blue squares. Sections are stained with HE. (A) Sagittal section of the posterior wall. (B) Schema of muscular structures around the anal canal in the posterior wall. (C) Frontal section of the lateral wall. (D) Schema of muscular structures around the anal canal in the lateral and anterolateral walls. The HL in the posterior wall and EF in the lateral wall branch from the LM. Longitudinal fibres (arrowheads) branch from the LM and run through intramuscular lesions of the EAS. CM, circular muscle; EAS, external anal sphincter; EF, endopelvic fascia; HE, haematoxylin and eosin; HL, hiatal ligament; IAS, internal anal sphincter; LAM, levator ani muscle; LM, longitudinal muscle; M, mucosa; MR, mesorectum; R, rectum; SM, submucosa

**FIGURE 2:**
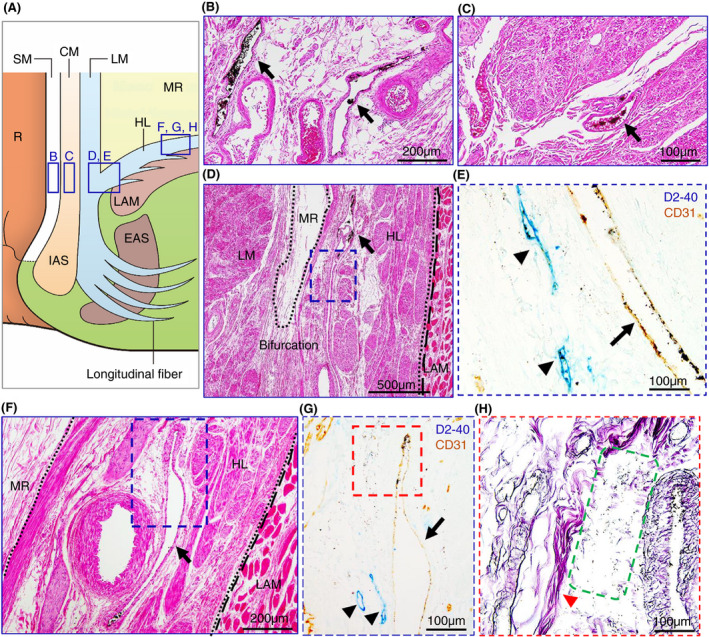
Enlarged views of India ink injected specimens around the LAM from the posterior wall. (A) Schema of the muscular structures. (B), (C), (D), (F) Enlarged views of HE staining in the SM (B), CM (C), bifurcation between the LM and HL (D) and HL (F). Veins with ink absorption are observed in all layers (arrows). (E), (G) Double‐stained immunohistochemistry for D2‐40 (lymphatic ducts, blue) and CD31 (blood vessels, brown). (H) Silver impregnation staining. (E) Enlarged view of the dashed blue square in (D). (G) Enlarged view of the dashed blue square in (F). (H) Enlarged view of the dashed red square in (G). In the bifurcation between the LM and HL (E) and in the HL (G), initial lymphatics (arrowheads) are observed around veins with ink absorption (arrows). In the HL, India ink can be observed in the perivenous interstitial space, indicated by the green dashed square in (H). Collagen fibres, indicated by the red arrowhead in (H), are also observed in this space. CM, circular muscle; EAS, external anal sphincter; HE, haematoxylin and eosin; HL, hiatal ligament; IAS, internal anal sphincter; LAM, levator ani muscle; LM, longitudinal muscle; MR, mesorectum; R, rectum; SM, submucosa

**FIGURE 3:**
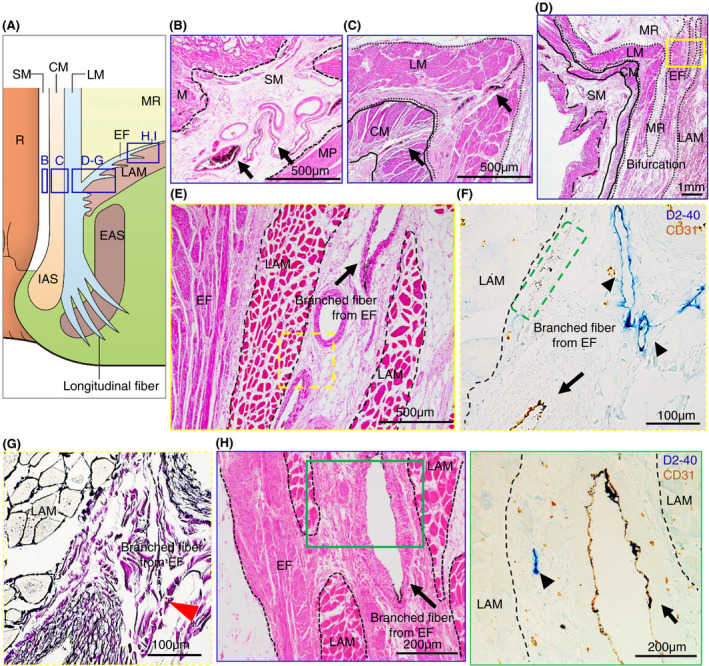
Enlarged views of India ink injected specimens around the LAM from the lateral and anterolateral wall. (A) Schema of the muscular structures. (B), (C), (D), (E), (H) HE staining in the SM (B), CM and LM (C), bifurcation between the LM and EF (D), (E) and EF (H). (E) Enlarged view of the yellow square in (D). Veins with ink absorption are observed in all layers (arrows). (F), (I) Double‐stained immunohistochemistry for D2‐40 (blue) and CD31 (brown). (F) Enlarged view of the yellow dashed square in (E). (I) Enlarged view of the green square in (H). (G) Silver impregnation staining for the yellow dashed square in (E). In the bifurcation between the LM and EF (E), (F) and in the EF (I), initial lymphatics (arrowheads) are observed around veins with ink absorption (arrows). In the branched fibres from the EF, India ink is observed in the perivenous interstitial space indicated by the green dashed square in (F). Collagen fibres indicated by the red arrowhead in (G) are also observed in this space. CM, circular muscle; EAS, external anal sphincter; EF, endopelvic fascia; HE, haematoxylin and eosin; IAS, internal anal sphincter; LAM, levator ani muscle; LM, longitudinal muscle; M, mucosa; MP, muscularis propria; MR, mesorectum; R, rectum; SM, submucosa

**FIGURE 4:**
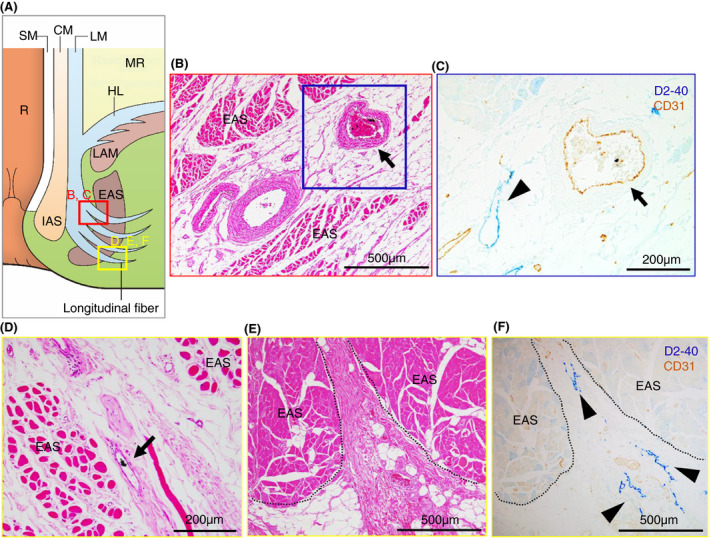
Enlarged views of India ink injected specimens around the EAS from the posterior, lateral and anterolateral walls. (A) Schema of the muscular structures around the EAS. (B), (C) Enlarged views of HE staining (B) and double‐stained immunohistochemistry for D2‐40 (blue) and CD31 (brown) (C) in the bifurcation between the LM and longitudinal fibres. (D), (E), (F) Enlarged views of HE staining (D), (E) and double‐stained immunohistochemistry (F) in intramuscular lesions of the EAS. In both areas, veins with ink absorption (arrow) and initial lymphatics (arrowheads) are observed. Lymphovascular structures around the EAS are similar in all walls. CM, circular muscle; EAS, external anal sphincter; EF, endopelvic fascia; HE, haematoxylin and eosin; HL, hiatal ligament; IAS, internal anal sphincter; LAM, levator ani muscle; LM, longitudinal muscle; MR, mesorectum; R, rectum; SM, submucosa

Our microanatomical examination of the human anorectal region disclosed the characteristic organization of connective tissue fasciae associated with the intestinal muscle layer, the LAM and EAS. Smooth muscle bundles with surrounding stromal tissue derived from the anorectal longitudinal muscle layer (LM) reached both posterior and bilateral surfaces of the LAM and EAS (Figure 1). The LM‐derivative tissue bundles diverged into the fascia around the LAM, referred to as the hiatal ligament (HL) in posterior components and the endopelvic fascia (EF) in lateral and anterolateral components. Another tissue bundle extended caudally from the LM, penetrated EAS muscles, and fused into the endomysium within the sphincter. The tissue bundles contained smooth muscle fibres, connective tissues, as well as abundant blood and lymphatic vessels (Figures 2, 3, 4).

India ink injections into the anal submucosal connective tissue combined with immunohistochemistry enabled the tracing of alternative tissue fluid flow from the anal canal. The ink penetrated anal venous vessels beyond our expectations. The deposition of India ink was detected in several venous lumina and their surrounding stroma in the rectal wall and fascia around the LAM from the junction of the rectal LM layer with the LAM fascia to the HL and EF (Figures 2 and 3). The stromal tissues around the ink‐storing blood vessels consisted of a reticular meshwork with collagen fibrils and contained several lymphatic vessels with podoplanin‐positive immunoreactivity (Figures 2H and 3G). India ink deposition into the venous lumina occurred in two cases (cases 1 and 7 in Table 1).

**FIGURE 5:**
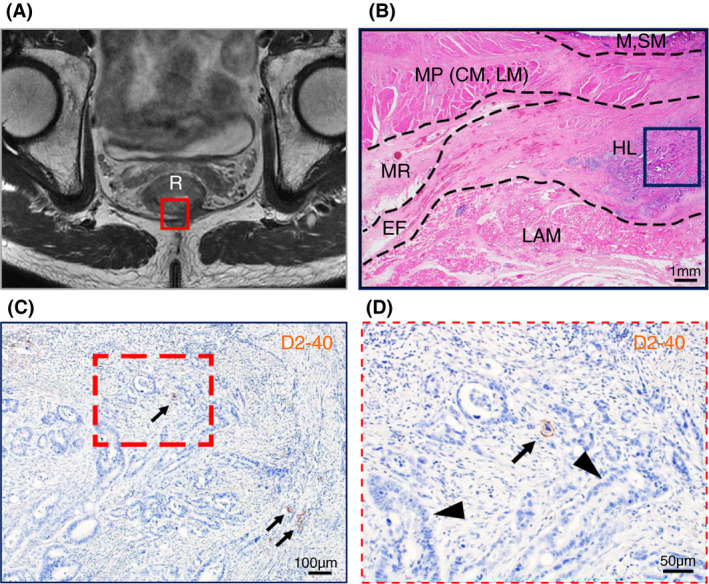
Magnetic resonance image and enlarged views of resected specimens of APR in a 57‐year‐old man with lower RC (case 2 in Table 1). (A) Axial magnetic resonance image. Tumour invasion to the LAM is suspected in the posterior portion. (B) Enlarged view of the HE staining for the axial section from the posterior wall to the right lateral wall (corresponding to the red square in (A)). Tumour cells are observed in the HL (blue square). (C) Enlarged view of the blue square in (B) stained by immunohistochemistry for D2‐40 (brown). Lymphatic ducts are observed in the HL (arrows). (D) Enlarged view of the red dashed square in (C). Tumour cells are observed in both stromal tissues (arrowheads) and lymphatic ducts (arrow). APR, abdominoperineal resection; CM, circular muscle; EF, endopelvic fascia; HE, haematoxylin and eosin; HL, hiatal ligament; LAM, levator ani muscle; LM, longitudinal muscle; M, mucosa; MP, muscularis propria; MR, mesorectum; RC, rectal cancer; R, rectum; SM, submucosa

Tumour invasion into the stromal tissue and lymphatic ducts in the HL was observed on the pathological sections (Figure 5). The EAS revealed ink deposition in some venous lumina from its confluent area with the LM‐extending tissue bundles (Figure 4).

**FIGURE 6:**
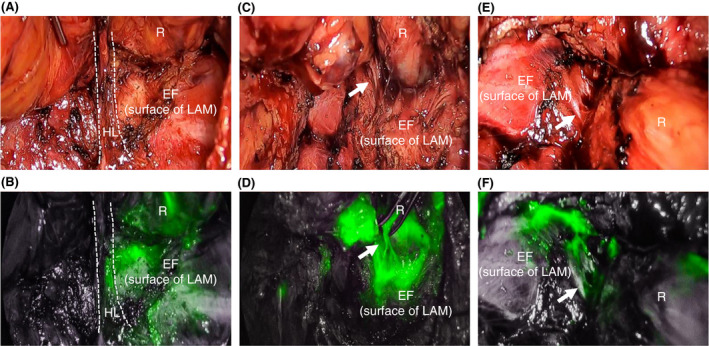
ICG‐FI findings in a 57‐year‐old man with lower RC (case 2 in Table 2). (A), (B) Images of the posterior wall before dissecting the HL (surrounded by dotted lines). (C), (D) Images of the posterior wall after dissecting the HL. (E), (F) Images of the left lateral and anterolateral wall. (B) Lymphatic flow (green) is observed in the deep part of the HL. (D), (F) Lymphatic flow is observed from muscular layers of the anal canal to the surface of the LAM (endopelvic fascia) through longitudinal muscle fibres (white arrow). (B), (D), (F) Near‐infrared images. EF, endopelvic fascia; HL, hiatal ligament; ICG‐FI, indocyanine green fluorescence imaging; LAM, levator ani muscle; R, rectum

ICG‐FI further demonstrated alternative tissue fluid flow in the intrapelvic spaces of the patients (Figure 6). ICG fluorescence was detected in both the caudal portion of the HL at the dorsal site (Figure 6A,B) and the EF at lateral and/or anterolateral sites (Figure 6C–F) with band‐like structures connecting the anorectal muscles.

## DISCUSSION AND CONCLUSIONS

This study investigated the transport of tissue fluid and substances within the human anal canal by microanatomical dissection using India ink injections combined with immunohistochemistry and intra‐operative ICG‐FI. We focused on tissue channels based on the fascial organization in the anorectal region to develop appropriate therapeutic strategies for lower RC.

**FIGURE 7:**
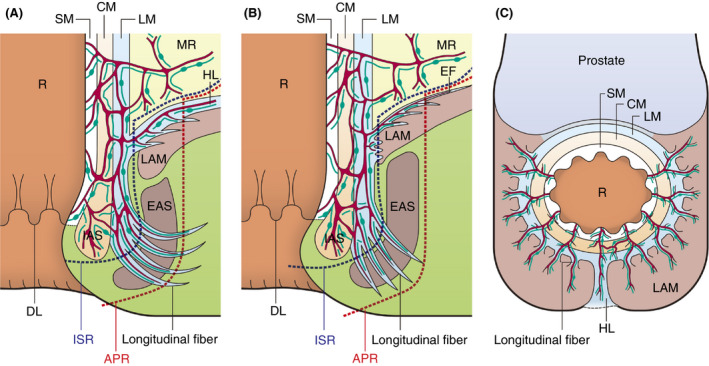
Schemas of lymphovascular flow around the anal canal. (A) Sagittal section of the posterior wall. (B) Frontal section of the lateral and anterolateral wall. (C) Male axial section around the anal canal and LAM. Communications of blood vessels (red solid lines) and initial lymphatics (green solid lines with round dots) from the LM to the HL, EF and longitudinal fibres are demonstrated. Resection lines of ISR (blue dotted line) and standard APR (red dotted line) are shown in (A) and (B). APR, abdominoperineal resection; CM, circular muscle; DL, dentate line; EAS, external anal sphincter; EF, endopelvic fascia; HL, hiatal ligament; IAS, internal anal sphincter; ISR, intersphincteric resection; LAM, levator ani muscle; LM, longitudinal muscle; MR, mesorectum; R, rectum; SM, submucosa

Our microanatomical examinations demonstrated an extensive musculostromal framework connecting the anorectal muscle coat to fascia spreading dorsolaterally on LAM and to EAS (Figure 7). The framework generally developed blood and lymphatic vascular networks indicating that the anorectal canal cooperates with pelvic parietal organs to build a functional complex structure with proper vasculature. The detection of ink in anal submucosal veins indicates prompt inflow into veins in the LAM fascia and the EAS epimysium/perimysium, implying that the anorectal‐pelvic framework establishes a peculiar circulation route. The injected ink was exclusively disseminated in the stromal tissue around blood vessels throughout the pelvic framework. This suggests that the perivascular stromal tissue serves as an essential drainage pathway for tissue fluid, certain substances, metabolites and motile cells, such as immune and tumour cells, from the anal canal. The histological composition of this perivascular space featuring reticular tissue such as that in lymphoid organs, draining tissue fluid and various immune cells is also considered adequate as a tissue channel. Previous studies reported that lymphatic vessels absorb interstitial fluid in the extracellular matrix, which consists of reticular and collagen fibres, thereby enabling lymphatic invasion [22, 23]. The ICG‐FI results of this study revealed that ICG flow throughout the LAM fascia shortly after dye injection into the anorectal submucosa in living humans coincided with the cadaveric data and supported the above concept. We also found that perivascular stromal tissue and lymphatic invasion were the actual tumour‐spreading pathway in patients with lower RC (Figure 5).

The present data exhibited a dense distribution of lymphatic vessels around blood vessels in the framework, but lymphatic vessels did not contain ink deposits in their lumina. This may be attributed to the functional collapse of lymphatics in the cadaveric and resected specimens. The injection of lymphatic tracers into the submucosa of the colon in fresh cadavers is well accepted [24, 25]. India ink is commonly used as a lymphatic tracer in fresh cadavers [26] but has also been injected into the submucosa of the colon in living subjects [27, 28]. Thus, India ink injection is a suitable method. However, unsatisfactory tissue preservation in cadaveric and resected specimens, such as shrinkage by 10% formaldehyde fixation and degeneration of tissue, may affect the environment of the lymphatic ducts. A previous report used low‐concentration formaldehyde to fix the specimen [26], which may affect the results. Although the ICG‐FI was unable to detect distinct vascular configurations in the patients in this study in whom lymphatic vessels are naturally capable of dye absorption and transport, the fascia around the LAM was thoroughly illuminated shortly after dye injection. Thus, stromal fluid, containing various substances and motile cells, may be primarily transported through the perivascular space rather than lymphatic vasculature and may subsequently flow from this prelymphatic channel into the lymphatics at certain sites in the fascial framework.

Based on our results, we discuss the therapeutic strategies for lower RC, especially in APR‐indicated cases. Our anatomical findings suggest a widespread lymphovascular network and tissue drainage beyond the resection line in APR (Figure 7A,B). Therefore, even if CRM involvement is negative based on APR, tumour cells may remain in the vasculature and stromal framework, especially in cases with positive lymphovascular invasion. This anatomical feature supports previous results suggesting that APR worsens the prognosis [2, 3, 4]. Based on our results, the ELAPE resection area includes the EF and HL, supporting the claims that ELAPE improves oncological outcomes. Accordingly, West et al. [6] demonstrated that ELAPE reduced CRM‐positive rates compared to APR (49.6% vs. 20.3%, *P* < 0.001). Lehtonen et al. [8] showed that ELAPE for T3–4 lower RC reduced local recurrence rates (6.7% vs. 15.5%, *P* = 0.048). Shen et al. [9] reported that ELAPE improved overall survival (41.5 months vs. 29.8 months, *P* = 0.028) and disease‐free survival (38.5 months vs. 29.3 months, *P* = 0.027). In contrast, Prytz et al. [11] reported that ELAPE had a high 3‐year risk of local recurrence (odds ratio 4.10, 95% confidence interval 1.19–14.08). However, the report by Prytz et al. included a selection bias, as the tumours were located more distally in the ELAPE group compared to the APR group (median distance from anal verge: ELAPE 3.0 cm vs. APR 6.0 cm). Inguinal lymph node (ILN) metastasis may occur in lower RC. Invasion of the dentate line and lymphovascular invasion are considered risk factors for ILN metastasis [29, 30]. However, microanatomical ILN metastatic pathways from the anorectal lesion have not been reported. Although our study did not disclose tumour translymphatic metastatic pathways from the anal canal to ILNs and the lateral pelvic compartment, particular lymphatic channels along the HL and EF may be associated with lymph node metastasis. One hypothesis to explain the failure of ELAPE to improve prognosis is the existence of a widespread functional lymphovascular network between anorectal lesions and these areas. Future research should expand the study area to inguinal and lateral pelvic lesions.

In addition, our study may provide important data regarding therapeutic strategies following local excision (LE). LE is an optional treatment for early lower RC [31, 32]. Although quality of life following LE is satisfactory, several studies have reported higher recurrence rates compared to total mesorectal excision [32, 33, 34, 35]. This can be explained by our microanatomical data which indicate that tumour cells invading the submucosal space in anorectal regions can spread widely through the HL and EF (Figure 7). To improve recurrence rates, LEs with adjuvant radiotherapy and chemoradiotherapy have been attempted [36, 37]. A study suggested the efficacy of adjuvant chemoradiotherapy for high‐risk early lower RC, defined as >1000‐µm submucosal invasion and the presence of lymphovascular invasion [38]. Currently, adjuvant chemoradiotherapy following LE is an optional treatment for early RC according to the guidelines [39].

This study is not without limitations. The sample size was small. In the study period, only seven participants and four participants were suitable for ink injection methods and ICG‐FI, respectively. Larger studies should be conducted to verify our results. Also, the anterior portion was not analysed in this study because the relationship between the anal canal and urogenital organs cannot be analysed in surgical specimens of APR. Discrepancies in the results of India ink injection and ICG‐FI may be due to the difference in the environment of tissue between specimens and living humans. The microanatomical data from resected specimens and ICG‐FI data were collected from patients with RC. Cancer induces lymphangiogenesis [40], and this might cause anatomical differences. Therefore, data collected from patients with RC might have a degree of uncertainty.

The study area was limited to the APR resection area. To discuss in more detail the therapeutic effects of ELAPE investigating the lymphatic pathways leading to lateral pelvic and inguinal lesions would be beneficial. Therefore, future studies that investigate lymphatic pathways in these areas are expected. To examine these areas, a combination of ICG‐FI and microanatomical analyses with fluorescent staining using fresh cadavers may be beneficial. This method may reduce the dissociation between macroscopic and microanatomical findings and allow for the observation of anorectal lymphatic flow, including the flow to lateral pelvic and inguinal lesions.

Our results suggest widespread functional lymphovascular flow pathways and tissue drainage around the anorectal region along the fascia on the surfaces of the LAM, HL and EF. Our early‐stage investigations may provide information to discuss therapeutic strategies for early and advanced lower RC.

## CONFLICT OF INTERESTS

The authors declare that they have no conflicts of interest.

## AUTHOR CONTRIBUTIONS

KS, HS, TM, YS, HM, SW, HN, YM, KU and KH contributed to the study concept and design. KS, HS, TM, SW, HN, YM and KU performed India ink injections in cadavers, resections of specimens from cadavers, and histological analyses. YS, HM, TM and KS performed the operations and intra‐operative ICG‐FI. KS and TM performed India ink injection in surgical specimens. HS and KH supervised the study. KS, HS, TM, YS, HM, SW, HN, YM, UM and KH participated in the interpretation of the results and the writing of the report. All authors read and approved the final manuscript.

## ETHICAL APPROVAL

This study was approved by the Human Research Ethics Committee of the Hirosaki University Graduate School of Medicine (Aomori, Japan; reference no. 2017‐1140, 2017‐044 and 2018‐134).

## CONSENT TO PARTICIPATE

Informed consent was obtained from all individual participants included in the study.

## CONSENT FOR PUBLICATION

The participants have consented to the submission of the descriptive study to a journal.

## Data Availability

The data that support the findings of this study are available on request from the corresponding author. The data are not publicly available due to privacy or ethical restrictions.

## References

[1] Okamura R , Hida K , Yamaguchi T , Akagi T , Konishi T , Yamamoto M , et al. Local control of sphincter‐preserving procedures and abdominoperineal resection for locally advanced low rectal cancer: propensity score matched analysis. Ann Gastroenterol Surg. 2017;1:199–207. 10.1002/ags3.12032 29863157 PMC5881346

[2] Law WL , Chu KW . Abdominoperineal resection is associated with poor oncological outcome. Br J Surg. 2004;91:1493–9. 10.1002/bjs.4723 15455362

[3] Silberfein EJ , Kattepogu KM , Hu CY , Skibber JM , Rodriguez‐Bigas MA , Feig B , et al. Long‐term survival and recurrence outcomes following surgery for distal rectal cancer. Ann Surg Oncol. 2010;17:2863–9. 10.1245/s10434-010-1119-8 20552409 PMC3071558

[4] Shihab OC , Brown G , Daniels IR , Heald RJ , Quirke P , Moran BJ . Patients with low rectal cancer treated by abdominoperineal excision have worse tumors and higher involved margin rates compared with patients treated by anterior resection. Dis Colon Rectum. 2010;53:53–6. 10.1007/DCR.0b013e3181c70465 20010351

[5] Holm T , Ljung A , Häggmark T , Jurell G , Lagergren J . Extended abdominoperineal resection with gluteus maximus flap reconstruction of the pelvic floor for rectal cancer. Br J Surg. 2007;94:232–8. 10.1002/bjs.5489 17143848

[6] West NP , Anderin C , Smith KJ , Holm T , Quirke P . Multicentre experience with extralevator abdominoperineal excision for low rectal cancer. Br J Surg. 2010;97:588–99. 10.1002/bjs.6916 20186891

[7] Han JG , Wang ZJ , Wei GH , Gao ZG , Yang Y , Zhao BC . Randomized clinical trial of conventional versus cylindrical abdominoperineal resection for locally advanced lower rectal cancer. Am J Surg. 2012;204:274–82. 10.1016/j.amjsurg.2012.05.001 22920402

[8] Lehtonen T , Rasanen M , Carpelan‐Holmstrom M , Lepisto A . Oncological outcomes before and after the extralevator abdominoperineal excision era in rectal cancer patients treated with abdominoperineal excision in a single centre, high volume unit. Colorectal Dis. 2019;21:183–90. 10.1111/codi.14468 30411461

[9] Shen Z , Bu Z , Li A , Lu J , Zhu L , Chong CS , et al. Multicenter study of surgical and oncologic outcomes of extra‐levator versus conventional abdominoperineal excision for lower rectal cancer. Eur J Surg Oncol. 2020;46:115–22. 10.1016/j.ejso.2019.08.017 31471089

[10] Asplund D , Haglind E , Angenete E . Outcome of extralevator abdominoperineal excision compared with standard surgery: results from a single centre. Colorectal Dis. 2012;14:1191–6. 10.1111/j.1463-1318.2012.02930.x 22221401

[11] Prytz M , Angenete E , Bock D , Haglind E . Extralevator abdominoperineal excision for low rectal cancer—extensive surgery to be used with discretion based on 3‐year local recurrence results: a registry‐based, observational national cohort study. Ann Surg. 2016;263:516–21. 10.1097/SLA.0000000000001237 25906414 PMC4741394

[12] Carpelan A , Karvonen J , Varpe P , Rantala A , Kaljonen A , Grönroos J , et al. Extralevator versus standard abdominoperineal excision in locally advanced rectal cancer: a retrospective study with long‐term follow‐up. Int J Colorectal Dis. 2018;33:375–81. 10.1007/s00384-018-2977-y 29445870

[13] Tsukada Y , Ito M , Watanabe K , Yamaguchi K , Kojima M , Hayashi R , et al. Topographic anatomy of the anal sphincter complex and levator ani muscle as it relates to intersphincteric resection for very low rectal disease. Dis Colon Rectum. 2016;59:426–33. 10.1097/DCR.0000000000000565 27050605

[14] Muro S , Tsukada Y , Harada M , Ito M , Akita K . Anatomy of the smooth muscle structure in the female anorectal anterior wall: convergence and anterior extension of the internal anal sphincter and longitudinal muscle. Colorectal Dis. 2019;21:472–80. 10.1111/codi.14549 30614646 PMC6850065

[15] Topor B , Acland R , Kolodko V , Galandiuk S . Mesorectal lymph nodes: their location and distribution within the mesorectum. Dis Colon Rectum. 2003;46:779–85. 10.1007/s10350-004-6656-4 12794580

[16] Miscusi G , di Gioia CR , Patrizi G , Gravetz A , Redler A , Petrozza V . Anatomical lymph node mapping in normal mesorectal adipose tissue. Dis Colon Rectum. 2010;53:1640–4. 10.1007/DCR.0b013e3181f48f90 21178858

[17] Bartholdson L , Hultborn A , Hultén L , Roos B , Rosencrantz M , Ahrén C . Lymph drainage from the upper and middle third of the rectum as demonstrated by ^198^Au. Acta Radiol Ther Phys Biol. 1977;16:352–60. 10.3109/02841867709133955 930639

[18] Takemura K , Ando M , Okabe S , Wakayama H , Jin KS , Ishii K , et al. Lymphatic drainage from the lower rectum. Nippon Daicho Komonbyo Gakkai Zasshi. 1986;39:113–20.

[19] Nishimura G , Yamaguchi A , Ishida T , Tani S , Yabushita K , Kato M , et al. Lymphatic drainage from the rectum as demonstrated by double isotope method. Nippon Daicho Komonbyo Gakkai Zasshi. 1990;43:153–8.

[20] Brierley JD , Gospodarowicz MK , Wittekind C . TNM Classification of Malignant Tumours, 8th ed. Oxford: John Wiley & Sons, Ltd, 2017.

[21] Watanabe J , Ota M , Suwa Y , Ishibe A , Masui H , Nagahori K . Evaluation of lymph flow patterns in splenic flexural colon cancers using laparoscopic real‐time indocyanine green fluorescence imaging. Int J Colorectal Dis. 2017;32:201–7. 10.1007/s00384-016-2669-4 27695977

[22] Wiig H , Keskin D , Kalluri R . Interaction between the extracellular matrix and lymphatics: consequences for lymphangiogenesis and lymphatic function. Matrix Biol. 2010;29:645–56. 10.1016/j.matbio.2010.08.001 20727409 PMC3992865

[23] Shimoda H , Kato S . A model for lymphatic regeneration in tissue repair of the intestinal muscle coat. Int Rev Cytol. 2006;250:73–108. 10.1016/S0074-7696(06)50003-8 16861064

[24] Jamieson JK , Dobson JF . The lymphatics of the colon. Proc R Soc Med. 1909;2:149–74. 10.1177/003591570900201506 PMC2046471 19974051

[25] Jamieson JK , Dobson JF . On the injection of lymphatics by Prussian blue. J Anat Physiol. 1910;45:7–10. 17232866 PMC1288882

[26] Clark RR , Shaw‐Dunn J , Soutar DS . A cadaveric study of auricular lymphatics and implications for sentinel lymph node biopsy. Clin Anat. 2010;23:792–7. 10.1002/ca.21015 20641070

[27] Kang J , Park HS , Kim IK , Song Y , Baik SH , Sohn SK , et al. Effect of preoperative colonoscopic tattooing on lymph node harvest in T1 colorectal cancer. Int J Colorectal Dis. 2015;30:1349–55. 10.1007/s00384-015-2308-5 26152843

[28] Aldecoa I , Montironi C , Planell N , Pellise M , Fernandez‐Esparrach G , Gines A , et al. Endoscopic tattooing of early colon carcinoma enhances detection of lymph nodes most prone to harbor tumor burden. Surg Endosc. 2017;31:723–33. 10.1007/s00464-016-5026-3 27324339 PMC5266760

[29] Wang R , Wu P , Shi D , Zheng H , Huang L , Gu W , et al. Risk factors of synchronous inguinal lymph nodes metastasis for lower rectal cancer involving the anal canal. PLoS One. 2014;9:e111770. 10.1371/journal.pone.0111770 25409168 PMC4237326

[30] Shiratori H , Nozawa H , Kawai K , Hata K , Tanaka T , Kaneko M , et al. Risk factors and therapeutic significance of inguinal lymph node metastasis in advanced lower rectal cancer. Int J Colorectal Dis. 2020;35:655–64. 10.1007/s00384-020-03520-2 32009191

[31] Steele GD Jr , Herndon JE , Bleday R , Russell A , Benson A , Hussain M , et al. Sphincter‐sparing treatment for distal rectal adenocarcinoma. Ann Surg Oncol. 1999;6:433–41. 10.1007/s10434-999-0433-5 10458680

[32] Garcia‐Aguilar J , Mellgren A , Sirivongs P , Buie D , Madoff RD , Rothenberger DA . Local excision of rectal cancer without adjuvant therapy: a word of caution. Ann Surg. 2000;231:345–51. 10.1097/00000658-200003000-00007 10714627 PMC1421005

[33] Mellgren A , Sirivongs P , Rothenberger DA , Madoff RD , García‐Aguilar J . Is local excision adequate therapy for early rectal cancer? Dis Colon Rectum. 2000;43:1064–74. 10.1007/BF02236551 10950004

[34] Paty PB , Nash GM , Baron P , Zakowski M , Minsky BD , Blumberg D , et al. Long‐term results of local excision for rectal cancer. Ann Surg. 2002;236:522–30. 10.1097/00000658-200210000-00015 12368681 PMC1422607

[35] Nash GM , Weiser MR , Guillem JG , Temple LK , Shia J , Gonen M , et al. Long‐term survival after transanal excision of T1 rectal cancer. Dis Colon Rectum. 2009;52:577–82. 10.1007/DCR.0b013e3181a0adbd 19404055

[36] Russell AH , Harris J , Rosenberg PJ , Sause WT , Fisher BJ , Hoffman JP , et al. Anal sphincter conservation for patients with adenocarcinoma of the distal rectum: long‐term results of radiation therapy oncology group protocol 89–02. Int J Radiat Oncol Biol Phys. 2000;46:313–22. 10.1016/s0360-3016(99)00440-x 10661337

[37] Rackley TP , Ma RM , Brown CJ , Hay JH . Transanal local excision for patients with rectal cancer: can radiation compensate for what is perceived as a nondefinitive surgical approach? Dis Colon Rectum. 2016;59:173–8. 10.1097/DCR.0000000000000544 26855390

[38] Sasaki T , Ito Y , Ohue M , Kanemitsu Y , Kobatake T , Ito M , et al. Postoperative chemoradiotherapy after local resection for high‐risk T1 to T2 low rectal cancer: results of a single‐arm, multi‐institutional, phase II clinical trial. Dis Colon Rectum. 2017;60:914–21. 10.1097/DCR.0000000000000870 28796729 PMC5553237

[39] Benson AB , Venook AP , Al‐Hawary MM , Cederquist L , Chen YJ , Ciombor KK , et al. Rectal cancer, version 2.2018, NCCN clinical practice guidelines in oncology. J Natl Compr Canc Netw. 2018;16:874–901. 10.6004/jnccn.2018.0061 30006429 PMC10203817

[40] Jakob C , Aust DE , Liebscher B , Baretton GB , Datta K , Muders MH . Lymphangiogenesis in regional lymph nodes is an independent prognostic marker in rectal cancer patients after neoadjuvant treatment. PLoS One. 2011;6:e27402. 10.1371/journal.pone.0027402 22087309 PMC3210168

